# Relationship of aging, skeletal muscle mass, and tooth loss with masseter muscle thickness

**DOI:** 10.1186/s12877-018-0753-z

**Published:** 2018-03-08

**Authors:** Kohei Yamaguchi, Haruka Tohara, Koji Hara, Ayako Nakane, Eriko Kajisa, Kanako Yoshimi, Shunsuke Minakuchi

**Affiliations:** Department of Gerodontology and Oral Rehabilitation, Tokyo Medical and Dental University, 1-5-45 yushima, Bunkyo-ku, Tokyo, 113-8510 Japan

**Keywords:** Aging, Elderly, Masseter muscle, Tooth loss, Skeletal muscle

## Abstract

**Background:**

Previous studies have reported a relationship between masseter muscle thickness and tooth loss or limb muscle thickness. However, it is not yet known whether masseter muscle thickness is related to appendicular skeletal muscle mass, and grip strength. The purpose of this study was to determine which of the two variables—tooth loss or appendicular skeletal muscle mass index—is more strongly related to masseter muscle thickness, and to identify a suitable indicator of decreasing masseter muscle thickness in healthy elderly individuals.

**Methods:**

Grip strength, walking speed, body weight, skeletal muscle mass index, tooth loss, and masseter muscle thickness at rest and during contraction were determined in 97 community-dwelling elderly individuals aged ≥65 years (men: 44, women: 53). Masseter muscle thickness was chosen as the dependent variable, while age, skeletal muscle mass index, body weight, grip strength, and tooth loss were chosen as the independent variables. Multiple regression analysis was conducted using the stepwise regression method.

**Results:**

In men, grip strength was the only independent predictor of masseter muscle thickness at rest. Tooth loss and grip strength were independent predictor of masseter muscle thickness during contraction. In women, tooth loss was the independent predictor of masseter muscle thickness both at rest and during contraction, while grip strength and body weight were the independent predictor of masseter muscle thickness at rest only.

**Conclusions:**

We confirmed that in healthy elderly individuals, tooth loss has a stronger relationship with masseter muscle thickness than aging and skeletal muscle mass index do. Masseter muscle thickness in both elderly men and women is also associated with grip strength, suggesting that grip strength can be used as an indicator of masseter muscle thickness in this population.

## Background

A generalized reduction in skeletal muscle mass and muscle strength occurs with aging. However, in elderly individuals, the influence of tooth loss on weakening of the masseter muscle—one of the masticatory muscles—must also be considered. A previous study showed that the masseter muscle is significantly thinner in an edentulous population than in a dentulous population [1]. Furthermore, the loss of masseter muscle mass due to tooth loss is well known [2].

Recent studies have reported a reduction in the strength of the perioral muscles due to sarcopenia [3]. Sarcopenia, due to aging and other reasons, results in a reduction in skeletal muscle mass, muscle strength, and physical function [4]. With regard to reduction in perioral muscle mass due to sarcopenia and malnutrition, a 2-year longitudinal study in elderly trauma patients showed the cross-sectional area of the masseter muscle to be a better predictor of 2-year mortality than the cross-sectional area of the psoas muscle. The psoas cross-sectional area is known as one of the objective indicators of sarcopenia, but cross-sectional area of the masseter muscle was shown to be a better predictive marker of sarcopenia in this study [5]. In addition, a cross-sectional study in 104 elderly individuals showed that tongue thickness decreases due to undernutrition in this population [6]. Another cross-sectional study in 197 community-dwelling elderly individuals showed that the reduction in perioral muscle strength due to sarcopenia results in reduced jaw opening force and tongue pressure [7]. The reduction in masseter muscle thickness (MMT) has also been shown to be related to a reduction in occlusal force and chewing ability [8], leading to difficulties in eating [9], and consequently, risk of malnutrition [10]. A previous study showed that 25% of community-dwelling elderly individuals were at risk of malnutrition [11]. Hence, it is important to ensure that the MMT is maintained in elderly individuals, so that masticatory function is preserved, thereby preventing malnutrition.

However, to date, it is unknown which one of the two factors—tooth loss or appendicular skeletal muscle mass and strength—have a stronger relationship with masticatory muscle mass and muscle strength. Identification of factors affecting MMT would enable the development of measures to prevent the weakening of the masseter muscle, thereby preserving masticatory function. Therefore, the aim of the present study was to determine which of the two variables—tooth loss or appendicular skeletal muscle mass index—is more strongly related to MMT, and to identify a suitable indicator of decreasing MMT in healthy elderly individuals.

## Methods

### Subjects

We enrolled 97 healthy community-dwelling elderly individuals aged ≥65 years. Two types of subject recruitment were conducted between December 2015 and August 2016. The first type of recruitment was conducted jointly by Tokyo Medical and Dental University and Oyama city (Tochigi, Japan), and involved volunteers who participated in a health survey targeting healthy elderly people in Oyama city. The second type of recruitment involved healthy elderly people who presented to the Gerodontics Clinic of the Dental Hospital at Tokyo Medical and Dental University for dental treatment. Of these elderly people, those with independent activities of daily living, who were able to follow instructions, and willing to participate, were included in this study. Those with a history of conditions that would affect muscle mass, such as neuromuscular diseases, were excluded. In addition, a previous study had shown that the cross-sectional area of the masseter muscle could be a marker of sarcopenia [5], so we excluded patients with sarcopenia to eliminate the effect of sarcopenia on MMT. Sarcopenia was diagnosed in accordance with the Asian Working Group for Sarcopenia algorithm [12]. Of a total of 128 participants, we excluded 21 because they were younger than 65 years, and another 10 because they had sarcopenia. We calculated the required sample size after estimating an effect size of 0.32 based on a previous research [6] using the G*Power3.1 software (Kiel University, Kiel, Germany). According to our calculations, using alpha = 0.05, power = 0.8, and effect size = 0.32, 43 patients would be required for the study. Informed consent was obtained from subjects for participation in the study, and the study was approved by the Tokyo Medical and Dental University Ethics Committee (ref: D2014–047). This research was a cross-sectional study, so participants were not followed up.

### Grip strength

Grip strength was measured using a hand dynamometer (TTM Inc., Chiba, Japan) with the subject in a standing position and their feet a shoulder-width apart. Measurements were taken twice in the dominant hand, and the larger value was taken as the final value.

### Walking speed

Subjects were instructed to walk along an 11-m walking path comprised of a 3-m acceleration path, a 5-m measurement path, and a 3-m deceleration path. We measured the time it took to walk from the start of the measurement path to the end. The walking speed was then calculated from these measurements.

### Skeletal muscle mass index

We measured appendicular muscle mass by bioimpedance analysis using In Body S10 (In Body Japan Inc., Tokyo, Japan). During measurements, subjects were instructed to rest in a seated position. The acquired appendicular muscle mass was divided by the square of the subject’s height and used as the skeletal muscle mass index (SMI).

### Tooth loss evaluation

Three dentists with at least 5 years of experience confirmed the condition of tooth loss through intraoral examination. The Eichner classification was used to classify patients in terms of tooth loss [13]. Subjects with all four regions of occlusal support in the right and left premolars, and the right and left molar regions were classified as the Eichner A group, those who had partial occlusal support in the premolar and molar region or occlusal support for the anterior teeth were classified as the Eichner B group, and subjects without any occlusal support were classified as the Eichner C group.

### Masseter muscle thickness

**Fig. 1:**
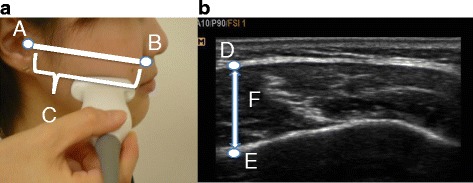
Masseter muscle thickness measurements. **a** Position of the probe during measurement. **b** Masseter muscle imaging with an ultrasonic diagnostic equipment. Upper edge of the tragus (B) Point under the nasal wing (C) Camper’s plane (D) Masseter muscle surface (E) Mandibular ramus (F) Masseter muscle thickness

MMT was measured by one dentist using the portable color ultrasound system, My Sono U6 (Samsung Madison Inc., Seoul, Korea). Subjects were instructed to sit, and the left and right MMTs were measured at rest and during contraction. The subjects were instructed to bite down as hard as possible for the measurements taken during contraction. We used a linear probe with a broadband frequency of 5–12 Mhz. Measurements were obtained at the midpoint between the zygomatic arch and mandibular angle, approximately parallel with the Camper’s plane along a line connecting the point under the nasal wing with the tragus of the ear, and the probe was placed perpendicular to the skin surface (Fig. 1). Measurements were taken twice, with a minimum of 2-min intervals between the first and the second measurements. Measurements were made using the stored image (Fig. 1). The thickest part on the image was determined [14], and the mean value of the right MMT was taken as the measurement. Intra-class correlation coefficients (ICCs) were calculated for measurements of MMT at rest and during contraction and the intra-rater reliability was assessed (Table 1). ICC (1,1) was the ICC for a single measurement by a single examiner, ICC (1,2) was the ICC for the mean of two measurements by a single examiner. ICC (1,2) exceeded 0.9, demonstrating a high intra-rater reliability.

**Table 1:** Intra-rater reliability of ultrasound measurements of the masseter muscle (*N* = 97)

	At rest	During contraction
ICC (1, 1)	0.83	0.86
95%CI	0.76–0.89	0.80–0.90
ICC (1,2)	0.91	0.92
95%CI	0.86–0.94	0.89–0.95

*ICC* Intra-class correlation coefficient

*95%CI* 95% confidence interval

### Statistical analysis

The normality of data was confirmed using the Shapiro-Wilk test. Tests for significance were then conducted using one-way analysis of variance and Kruskal-Wallis test between each category of the Eichner classification. Statistical significance was set at *P* < .05. In addition, Tukey’s honest significant difference test was used for multiple comparisons of MMT during contraction in men and MMT at rest in women.

MMT at rest and during contraction were set as the dependent variables, while age, SMI, body weight, grip strength, and tooth loss were the independent variables. Tooth loss was entered into the regression model as a categorical variable (Eichner A, B, or C). Multiple regression analysis was conducted using the stepwise regression method with analysis of variance to test for statistical significance. The correlation matrix table was confirmed in advance. Correlations between MMT and each parameter were assessed using Pearson’s correlation coefficients and Spearman’s rank correlation coefficients. There was a gender difference in the independent variables when the multiple regression analysis included both men and women; therefore, multiple regression analysis was conducted separately for men and women. Data were analyzed with SPSS version 21.0 J (IBM Inc., Tokyo, Japan).

## Results

**Fig. 2:**
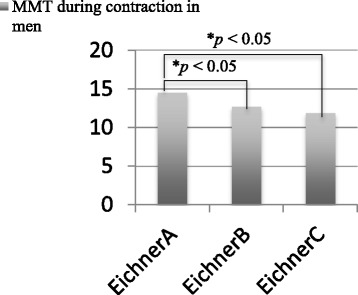
Statistical difference between masseter muscle thickness (MMT) during contraction in men in Tukey’s honest significant difference test

Subject characteristics are shown in Table 2. There were significant differences (*P* < .05) between different Eichner classifications in age and MMT during contraction in men, and in age, walking speed, and MMT at rest in women. Tukey’s honest significant difference test revealed significant differences between Eichner A and Eichner B, and between Eichner A and Eichner C in men (Fig. 2). There was no significant difference in MMT at rest in women in Tukey’s honest significant difference test.

**Table 2:** Subject Characteristics (N = 97)

	Eichner A	Eichner B	Eichner C	*P*-value
Men	*n* = 13	*n* = 25	*n* = 6	
Age, years, median (IQR)	68 (67.0_71.5)	77 (74.5_84.5)	74.5 (71.0_85.0)	< .01^a^
SMI, kg/m^2^, mean ± SD	7.5 ± 0.8	7.7 ± 0.9	8.0 ± 0.8	.49
Weight, kg, mean ± SD	61.2 ± 9.6	62.0 ± 10.8	66 ± 3.5	.60
Grip strength, kg, mean ± SD	35.2 ± 5.2	31.4 ± 6.3	36.2 ± 5.8	.09
Walking speed, m/s, median (IQR)	1.3 (1.2_1.5)	1.3 (1.1_1.4)	1.3 (1.2_1.5)	.30
MMT at rest, mm, mean ± SD	10.7 ± 2.0	9.6 ± 1.9	9.9 ± 1.6	.25
MMT during contraction, mm, mean ± SD	14.5 ± 2.3	12.7 ± 2.1	11.9 ± 1.4	< .05^b^
Women	*n* = 28	*n* = 20	*n* = 5	
Age, years, median (IQR)	70 (67.3_72.8)	73 (68.3_75.0)	78 (75.5_82.0)	< .01^a^
SMI, kg/m^2^, mean ± SD	6.2 ± 0.7	6.2 ± 0.7	6.0 ± 0.7	.84
Weight, kg, mean ± SD	53.0 ± 8.7	54.5 ± 7.1	48.6 ± 9.0	.36
Grip strength, kg, mean ± SD	23.8 ± 4.3	23.0 ± 3.8	22.1 ± 5.9	.65
Walking speed, m/s, median (IQR)	1.5 (1.3_1.6)	1.4 (0.9_1.4)	1.1 (0.8_1.5)	< .05^a^
MMT at rest, mm, mean ± SD	8.7 ± 1.5	8.0 ± 1.8	7.0 ± 1.2	< .05^b^
MMT during contraction, mm, mean ± SD	11.3 ± 1.7	10.4 ± 1.9	9.7 ± 1.8	.09

*MMT* masseter muscle thickness

^a^Kruskal-Wallis test, ^b^One-way analysis of variance, *SMI* skeletal muscle mass index,

The correlation between the variables was confirmed in Table 3, and none of the variables had r > .8. The results of the multiple regression analysis conducted separately for men and women using MMT at rest and during contraction as the dependent variables are shown in Table 4. The adjusted R^2^ was 0.3 or less; therefore, there was not a high degree of consistency. However, the data represented the extent to which independent variables affected dependent variables. Table 4 shows that the independent predictor of MMT at rest was grip strength in men, and tooth loss, grip strength, and whole-body weight in women. During muscle contraction, the independent variables were tooth loss and grip strength in men, and tooth loss only in women. A comparison of the standard partial regression coefficients of tooth loss (− 0.39, *P < 0.01*) and grip strength (0.32, *P = 0.03*) with MMT during contraction in men showed that tooth loss had a stronger relationship with MMT than did grip strength. A comparison of the standard partial regression coefficients of tooth loss (− 0.35, *P < 0.01)*, grip strength (− 0.27, *P = 0.05)*, and body weight (0.38, *P < 0.01)* with MMT at rest in women showed that tooth loss and body weight had a similar strength of association with MMT. In men, the relationship between grip strength and MMT was observed both at rest and during contraction, but in women, it was observed only at rest. For tooth loss, the relationship with MMT was observed both at rest and during contraction in women, but only during contraction in men.

**Table 3:** Correlation matrix table of parameters

	MMT during contraction	MMT at rest	Age	Body Weight	GS	SMI	Tooth loss: Eichner classification
Men							
MMT during contraction	1.00		−0.31^*^	0.14	0.34^*^	0.22	−0.41 ^*^
MMT at rest		1.00	−0.30^*^	0.10	0.33^*^	0.25	−0.18
Age			1.00	−0.20	−0.52^*^	− 0.17	0.49^*^
Body Weight				1.00	0.32^*^	0.74^*^	0.14
GS					1.00	0.27^*^	− 0.05
SMI						1.00	0.17
Women							
MMT during contraction	1.00		−0.21	0.18	0.11	0.10	−0.31^*^
MMT at rest		1.00	−0.25	0.30 ^*^	−0.08	0.21	−0.34^*^
Age			1.00	−0.27	−0.21	− 0.24	0.44^*^
Body weight				1.00	0.38^*^	0.73^*^	− 0.07
GS					1.00	0.38^*^	− 0.13
SMI						1.00	−0.03

^*^p-value < 0.05, *MMT* Masseter muscle thickness, *GS* Grip strength *SMI* Skeletal muscle mass index

**Table 4:** Multiple regression analysis values with masseter muscle thickness at rest and during contraction as the dependent variables

Dependent Variable: MMT	Independent Variable	Standard Partial Regression coefficient	*P* value	95% CI	R	Adjusted R^2^
At rest	Men					
	Grip strength	0.33	0.030	0.011–0.20	0.33	0.09
	Women					
	Tooth loss	−0.35	< .01	−1.48—0.24	0.50	0.21
	Grip strength	−0.27	0.050	−0.21-0.00		
	Body Weight	0.38	< .01	0.022–0.13		
During Contraction	Men					
	Tooth loss	−0.39	< .01	−2.32–0.43	0.52	0.24
	Grip strength	0.32	0.030	0.020–0.22		
	Women					
	Tooth loss	−0.31	0.027	−1.6—0.10	0.31	0.075

*95%CI* 95% confidence interval, *MMT* Masseter muscle thickness

## Discussion

We investigated the relationship of age, tooth loss, grip strength, and SMI with MMT in healthy elderly individuals. Our results indicate that MMT in men and women is associated with both tooth loss and grip strength. However, some gender differences were noted.

Although there is no previous report on the relationship between grip strength and MMT, a relationship between grip strength and occlusal force has been reported in 489 community-dwelling elderly individuals aged ≥85 years [15]. In another study, grip strength was shown to have a higher correlation with body skeletal muscle mass than did knee extension strength in 405 community-dwelling elderly individuals, suggesting that it could be used as a reasonable indicator of body skeletal muscle strength in this population [16]. We consider why grip strength and occlusal force in elderly individuals were related.

Aging is one of the factors that result in a decrease in muscle strength. In general, muscle mass and muscle strength decrease with age. A previous study reported that the muscle mass of an elderly man aged 70 years is approximately 15% less than that of an adult aged 20 years [17]. It has also been reported that not only limb muscle strength but also masticatory muscle strength decreases with aging [15]. This is believed to be due to the effect of the hormones insulin-like growth factor 1 (IGF-1) and testosterone. IGF-1 and testosterone are vital hormones for muscle enhancement and repair. Testosterone has been reported to be related not only to muscle mass but also to muscle strength [18], and a positive correlation between testosterone and grip strength has been reported [19]. Secretion of these hormones decreases with aging [20]. Furthermore, in a previous study using an experimental mouse model, the masseter muscle mass was shown to decrease after castration and increase by 38% upon testosterone supplementation; the response was more robust than that for limb muscle, suggesting a greater effect of testosterone on masseter muscle mass [21]. For these reasons, grip strength and occlusal force in elderly decrease similarly. Since it has been shown that MMT is strongly related to occlusal force [22], an association between MMT and grip strength was anticipated.

In this study, we also showed a relationship between tooth loss and MMT. In a previous study investigating the effect of tooth loss on MMT, the MMT in edentulous patients was observed to be significantly smaller than that in dentate patients [1]. In our research, among men, the MMT in individuals with Eichner C classification was significantly smaller than that in individuals with Eichner A. An increase in MMT due to dental treatment has also been reported previously. A study that compared MMT in edentulous patients immediately after fitting complete dentures with that after three months of use reported that the masseter muscle was significantly thicker after three months of denture wear [1]. It has also been shown that the MMT significantly increases with dental implant therapy [23]. Another study reported an increase in MMT after correction of the occlusal relationship through orthodontic treatment in teenagers [24]. However, these studies were not limited to elderly individuals, and they did not investigate the effect of appendicular skeletal muscle mass and muscle strength. Therefore, to date, it is unknown whether occlusal recovery is effective in increasing MMT and pathologically reduced skeletal muscle mass and muscle strength in elderly patients with sarcopenia.

When considering an increase in MMT in healthy elderly subjects due to dental treatment, it is important to consider the cause of muscle atrophy and the ratio of the type of muscle fiber constituting the masseter muscle. Muscle atrophy is caused by aging-related factors such as primary sarcopenia and muscle disuse. Muscle atrophy from disuse is reported to progress at a rate of approximately 10% after one month of bed rest [25], so this is one of the main causes of reduced muscle mass in elderly individuals. Muscle fiber atrophy associated with disuse is known to involve predominantly type I fibers [26], while aging involves predominantly type II fibers [27]. The muscle fiber structure of the masseter muscle contains both type I and type II fibers, but reports show that the fibers are mainly type I [28]. Therefore, reduction in masseter muscle mass is considered to be more strongly affected by inactivity rather than aging. Even in the present study, aging did not exhibit a relationship with MMT; instead, tooth loss had a stronger relationship with MMT than did aging and SMI. Therefore, if masseter muscle activity is enhanced through appropriate occlusal recovery [29], then an increase in MMT could be expected in healthy elderly individuals irrespective of their age.

This study has some limitations. First, the sample size was small, therefore, the effect size was low for MMT at rest in men, and MMT during contraction in women. Using a larger sample size may result in an increase in the effect size. Second, since this study was cross-sectional in design, it was not possible to investigate the effect on weakness of the MMT, such as sarcopenia. Third, we only investigated the muscle mass, and did not investigate muscle strength, i.e., occlusal force in the masseter muscle. In a future study, we intend to establish a group comprising patients with sarcopenia, and conduct a more detailed investigation of the relationship between perioral muscle mass and muscle strength. Finally, frailty could also be a confounding factor for MMT [30], but we have not been able to examine this in this study.

Despite the above limitations, this research provides a clinically significant result that grip strength can be used as an indicator of decrease in MMT, i.e., deterioration of masticatory efficiency [8]. Especially, in men, this relationship was observed both at rest and during contraction of the masseter muscle. In contrast, in women, tooth loss was observed to be associated with MMT both at rest and during contraction of the muscle. Deterioration of masticatory efficiency restricts nutrition intake, such as protein and carbohydrate intake [9], thereby increasing the risk of malnutrition [10]. Measurement of grip strength is an easy and noninvasive procedure, so it could be useful as a screening tool for decrease in MMT in hospitals as well the community.

## Conclusions

We confirmed that MMT in healthy elderly individuals is associated with grip strength. Our results suggest that the decrease in grip strength could be an indicator of decrease in masseter muscle mass, especially in men. Tooth loss has a stronger relationship with MMT than aging and SMI have, in both men and women. Therefore, it is possible to increase the MMT through occlusal recovery in healthy elderly individuals irrespective of their age.

## Abbreviations

ICC: Intra-class correlation coefficients
IGF-1: Insulin-like-growth factor 1
MMT: Masseter muscle thickness
SMI: Skeletal muscle mass index

## References

[1] Bhoyar PS, Godbole SR, Thombare RU, Pakhan AJ (2012). Effect of complete edentulism on masseter muscle thickness and changes after complete denture rehabilitation: an ultrasonographic study. J Investig Clin Dent.

[2] Newton JP, Abel EW, Robertson EM, Menhinick S (1993). Changes in human jaw muscles with age and dental state. Gerodontology.

[3] Maeda K, Akagi J (2015). Decreased tongue pressure is associated with sarcopenia and sarcopenic dysphagia in the elderly. Dysphagia.

[4] Cruz-Jentoft AJ, Baeyens JP, Bauer JM, Boirie Y, Cederholm T, Landi F (2010). Sarcopenia: European consensus on definition and diagnosis: report of the European working group on sarcopenia in older people. Age Ageing.

[5] Wallace JD, Calvo RY, Lewis PR, Brill JB, Shackford SR, Sise MJ (2017). Sarcopenia as a predictor of mortality in elderly blunt trauma patients: comparing the masseter to the psoas using computed tomography. J Trauma Acute Care Surg.

[6] Tamura F, Kikutani T, Tohara T, Yoshida M, Yaegaki K (2012). Tongue thickness relates to nutritional status in the elderly. Dysphagia.

[7] Machida N, Tohara H, Hara K, Kumakura A, Wakasugi Y, Nakane A (2016). Effects of aging and sarcopenia on tongue pressure and jaw-opening force. Geriatr Gerontol Int.

[8] Müller F, Hernandez M, Grütter L, Aracil-Kessler L, Weingart D, Schimmel M (2012). Masseter muscle thickness, chewing efficiency and bite force in edentulous patients with fixed and removable implant-supported prostheses: a cross-sectional multicenter study. Clin Oral Implants Res.

[9] Saarela RK, Lindroos E, Soini H, Hiltunen K, Muurinen S, Suominen MH (2016). Dentition, nutritional status and adequacy of dietary intake among older residents in assisted living facilities. Gerodontology.

[10] Kikutani T, Yoshida M, Enoki H, Yamashita Y, Akifusa S, Shimazaki Y (2013). Relationship between nutrition status and dental occlusion in community-dwelling frail elderly people. Geriatr Gerontol Int.

[11] Cuervo M, García A, Ansorena D, Sánchez-Villegas A, Martínez-González M, Astiasarán I (2009). Nutritional assessment interpretation on 22,007 Spanish community-dwelling elders through the mini nutritional assessment test. Public Health Nutr.

[12] Chen LK, Liu LK, Woo J, Assantachai P, Auyeung TW, Bahyah KS (2014). Sarcopenia in Asia: consensus report of the Asian working Group for Sarcopenia. J Am Med Dir Assoc.

[13] Eichner K (1955). Über eine Gruppeneintelung des Lückengebisses für die Prothetik. Dtsch Zahnärztl Z.

[14] Serra MD, Duarte Gavião MB, dos Santos Uchôa MN (2008). The use of ultrasound in the investigation of the muscles of mastication. Ultrasound Med Biol.

[15] Iinuma T, Arai Y, Fukumoto M, Takayama M, Abe Y, Asakura K (2012). Maximum occlusal force and physical performance in the oldest old: the Tokyo oldest old survey on total health. J Am Geriatr Soc.

[16] Yamada Y, Watanabe Y, Ikenaga M, Yokoyama K, Yoshida T, Morimoto T (2013). Comparison of single- or multifrequency bioelectrical impedance analysis and spectroscopy for assessment of appendicular skeletal muscle in the elderly. J Appl Physiol (1985).

[17] Gallagher D, Visser M, De Meersman RE, Sepúlveda D, Baumgartner RN, Pierson RN (1997). Appendicular skeletal muscle mass: effects of age, gender, and ethnicity. J Appl Physiol.

[18] Roy TA, Blackman MR, Harman SM, Tobin JD, Schrager M, Metter EJ (2002). Interrelationships of serum testosterone and free testosterone index with FFM and strength in aging men. Am J Physiol Endocrinol Metab.

[19] Mohr BA, Bhasin S, Kupelian V, Araujo AB, O'Donnell AB, McKinlay JB (2007). Testosterone, sex hormone-binding globulin, and frailty in older men. J Am Geriatr Soc.

[20] Albani D, Batelli S, Polito L, Vittori A, Pesaresi M, Gajo GB (2009). A polymorphic variant of the insulin-like growth factor 1 (IGF-1) receptor correlates with male longevity in the Italian population: a genetic study and evaluation of circulating IGF-1 from the “Treviso Longeva (TRELONG)” study. BMC Geriatr.

[21] Widmer CG, Morris-Wiman J (2010). Limb, respiratory, and masticatory muscle compartmentalization: developmental and hormonal considerations. Prog Brain Res.

[22] Raadsheer MC, van Eijden TM, van Ginkel FC, Prahl-Andersen B (1999). Contribution of jaw muscle size and craniofacial morphology to human bite force magnitude. J Dent Res.

[23] Gonçalves TM, Campos CH, Gonçalves GM, de Moraes M, Rodrigues Garcia RC (2013). Mastication improvement after partial implant-supported prosthesis use. J Dent Res.

[24] Kiliaridis S, Mahboubi PH, Raadsheer MC, Katsaros C (2007). Ultrasonographic thickness of the masseter muscle in growing individuals with unilateral crossbite. Angle Orthod.

[25] Berry P, Berry I, Manelfe C (1993). Magnetic resonance imaging evaluation of lower limb muscles during bed rest - a microgravity simulation model. Aviat Space Environ Med.

[26] Ciciliot S, Rossi AC, Dyar KA, Blaauw B, Schiaffino S (2013). Muscle type and fiber type specificity in muscle wasting. Int J Biochem Cell Biol.

[27] Lexell J, Taylor CC, Sjöström M (1988). What is the cause of the ageing atrophy? Total number, size and proportion of different fiber types studied in whole vastus lateralis muscle from 15- to 83-year-old men. J Neurol Sci.

[28] Osterlund C, Thornell LE, Eriksson PO (2011). Differences in fibre type composition between human masseter and biceps muscles in young and adults reveal unique masseter fibre type growth pattern. Anat Rec (Hoboken).

[29] von der Gracht I, Derks A, Haselhuhn K, Wolfart S. EMG correlations of edentulous patients with implant overdentures and fixed dental prostheses compared to conventional complete dentures and dentates: a systematic review and meta-analysis. Clin Oral Implants Res. 2016; 10.1111/clr.12874. 10.1111/clr.12874 27302014

[30] Watanabe Y, Hirano H, Arai H, Morishita S, Ohara Y, Edahiro A (2017). Relationship between frailty and oral function in community-dwelling elderly adults. J Am Geriatr Soc.

